# Phylogenetic analyses and characteristics of the microbiomes from five mealybugs (Hemiptera: Pseudococcidae)

**DOI:** 10.1002/ece3.4889

**Published:** 2019-01-21

**Authors:** Dan lin, Li Zhang, Weidong Shao, Xuelian Li, Xunyue Liu, Huiming Wu, Qiong Rao

**Affiliations:** ^1^ School of Agriculture and Food Science Zhejiang A & F University Hangzhou China; ^2^ Zhoushan Entry‐exit Inspection and Quarantine Breau Ningbo China; ^3^ Ningbo Entry‐exit Inspection and Quarantine Bureau Ningbo China

**Keywords:** coevolution, high‐throughput sequencing, mealybugs, symbiont diversity

## Abstract

Associations between Sternorrhyncha insects and intracellular bacteria are common in nature. Mealybugs are destructive pests that seriously threaten the production of agriculture and forestry. Mealybugs have evolved intimate endosymbiotic relationships with bacteria, which provide them with essential amino acids, vitamins, and other nutrients. In this study, the divergence of five mealybugs was analyzed based up the sequences of the mitochondrial cytochrome oxidase I (mtCOI). Meanwhile, the distinct regions of the 16S rRNA gene of primary symbionts in the mealybugs were sequenced. Finally, high‐throughput sequencing (HTS) techniques were used to study the microbial abundance and diversity in mealybugs. Molecular phylogenetic analyses revealed that these five mealybugs were subdivided into two different clusters. One cluster of mealybugs (*Dysmicoccus neobrevipes*, *Pseudococcus comstocki*, and *Planococcus minor*) harbored the primary endosymbiont “*Candidatus* Tremblaya princeps,” and another cluster (*Phenacoccus solenopsis *and *Phenacoccus solani*) harbored “*Ca*. Tremblaya phenacola.” The mtCOI sequence divergence between the two clusters was similar to the 16S rRNA sequence divergence between *T. princeps* and *T. phenacola*. Thus, we concluded that the symbiont phylogeny was largely concordant with the host phylogeny. The HTS showed that the microbial abundance and diversity within *P. solani* and *P. solenopsis *were highly similar, and there was lower overall species richness compared to the other mealybugs. Among the five mealybugs, we also found significant differences in Shannon diversity and observed species. These results provide a theoretical basis for further research on the coevolution of mealybugs and their symbiotic microorganisms. These findings are also useful for research on the effect of symbiont diversity on the pest status of mealybugs in agricultural systems.

## 1 INTRODUCTION

Hemiptera insects, such as aphids, scales, and whiteflies, feed solely on plant sap throughout their life (Baumann & Baumann, 2005). However, phloem is rich in carbohydrates and deficient in amino acids (Douglas, 1998). Therefore, mealybugs require the presence of microorganisms to provide additional nutrients (Downie & Gullan, 2004, 2005; Moran, Plague, Sandström, & Wilcox, 2003). In terms of their inseparability from insect survival, endosymbionts are further classified as primary endosymbionts (Abb. P‐endosymbiont) and secondary symbionts (Abb. S‐symbiont here) (Engel & Moran, 2013; Wernegreen & Wheeler, 2009). The dominant bacteria in bacteriocytes (generally, P‐endosymbionts) rarely experience interference from the external environment and can be transmitted vertically by hosts. Thus, the insects are able to precisely control the location and abundance of the endosymbionts (Gruwell, Hardy, Gullan, & Dittmar, 2010). S‐symbionts, which can be horizontally or vertically transferred, are not restricted only to bacteriocytes. Apart from their important roles in supplementing essential nutrients to insect, some endosymbionts can also play crucial roles in protecting hosts from damage by producing toxins and regulating the reproductive and immune systems of the insect (Moran, McCutcheon, & Nakabachi, 2008).

Mealybugs (Hemiptera: Pseudococcidae), a large family of scale insects (Coccoidea) including 259 genera and 1997 species (García Morales et al., 2016), cause serious threats to the production of agriculture and forestry (Wu, Ma, Hu, & Zeng, 2015). However, the morphological identification of mealybug species is difficult and limited by a high degree of similarity and polymorphism (especially in nymphs or eggs), which pose challenges in the study and management of these insects (Pacheco da Silva et al., 2014). The diagnostic PCR assay developed here provides a quick, simple, and reliable molecular technique for identifying and monitoring mealybugs, and this assay will be useful for intercepting and blocking the further spread of invasive mealybugs (Beltrà, Soto, & Malausa, 2012; Gruwell et al., 2010; Malausa et al., 2011). The mitochondrial cytochrome oxidase I (COI) gene has been demonstrated to be effective in studying the phylogenetic relationships of insects, for example, *Bemisia tabaci *(De Barro, Liu, Boykin, & Dinsdale, 2011; Rao, Luo, Zhang, Guo, & Devine, 2011). Likewise, this method has also been applied to the scale insects to construct biological and phylogenetic models, providing a theoretical basis for pest control (Abd‐Rabou et al., 2012; Correa, Germain, Malausa, & Zaviezo, 2012; Park, Suh, Hebert, Oh, & Hong, 2011; Saccaggi, Krüger, & Pietersen, 2008). This sequencing method has been widely used in biological species identification and genetic analysis. A recent study proposed that certain endosymbiotic Flavobacteria, which are considered to be primary endosymbionts of scale insects, codiversified with their Monophlebidae hosts (Von Dohlen, Kohler, Alsop, & McManus, 2001; Gruwell et al., 2010; Rosenblueth, Sayavedra, Samano‐Sanchez, Roth, & Martinez‐Romero, 2012). The primary endosymbionts, “*Candidatus* Tremblaya princeps,” are members of the β‐subdivision of the Proteobacteria, which are found in nearly all mealybug species and have a monophyletic origin (Baumann & Baumann, 2005; Downie & Gullan, 2005). The research showed that certain *Phenacoccus* species harbor another β‐proteobacterial endosymbiont, “*Candidatus* Tremblaya phenacola.” *T. phenacola* is a sister clade of *T. princeps*, suggesting that the Pseudococcidae share a common and single evolutionary source of the endosymbionts (Downie & Gullan, 2005; Gruwell et al., 2010), whereas the secondary γ‐proteobacterial endosymbionts, including *Moranella endobia* (Mccutcheon & Dohlen, 2011), are of polyphyletic evolutionary origins. The S‐symbiont clusters of 12 representative mealybug species were distinct from each other and from other insect‐associated bacteria (Thao, Gullan, & Baumann, 2002). A number of mealybugs harbor secondary γ‐proteobacterial endosymbionts, which are contained within the cell of the β‐proteobacteria, *T. princeps* (Von Dohlen et al., 2001). This unusual nested relationship between the β‐proteobacteria and γ‐proteobacteria has been well studied in recent research (Gatehouse, Sutherland, Forgie, Kaji, & Christeller, 2011; Kono, Koga, Shimada, & Fukatsu, 2008; Sergio, Amparo, Manuel, Andrés, & Rosario, 2013; Von Dohlen et al., 2001). For example, *T. princeps* in *Planococcus citri* (Risso) harbors the γ–proteobacterium “*Candidatus* Moranella endobia” (Lopez‐Madrigal et al., 2014). Through the intensive study of this nested structure, genome information revealed that *M. endobia* appears to be responsible for the biosynthesis of the majority of the cellular constituents and the energy supply, as well as the regulation of most informational courses for this special consortium, while *T. princeps *reserves the genes involved in indispensable informational functions (Lopez‐Madrigal et al., 2013).

Additionally, the β‐endosymbiotic genome has experienced a tremendous reduction due to the high incidence of gene drift and the relaxation of gene purification options (Sabatermuñoz, Toft, Alvarez‐Ponce, & Fares, 2017) Therefore, P‐endosymbionts are likely to lack the genes associated with the metabolic functions required by insects, but studies have also shown that these genes can be obtained from S‐symbionts that are nested within P‐endosymbionts (Wernegreen & Wheeler, 2009).

The 16S ribosomal RNA (especially the V3–V4 variable region) gene has been verified to be an accurate, reliable, and repeatable marker for symbiont identification and phylogenetic analysis, since the sequence is highly conserved and universally distributed (Degnan & Ochman, 2012). In addition, with the continuous development of high‐throughput sequencing platforms, HTS is considered to be a valid tool for researching microbial communities, which do not depend on previous knowledge about the diversity of the bacterial communities that are under investigation (Goodrich et al., 2014). These methods are based on the deep sequencing of PCR‐amplified bacterial 16S rRNA gene fragments, and the sequence data generated by HTS techniques are usually grouped into the same operational taxonomic unit (OTU) based on sequence similarity (≥97%), enabling the detection of all bacterial species existing in various samples. OTU is an effective tool for exploring bacterial communities, such as the symbiosis in aphids and psyllidae (Gauthier, Outreman, Mieuzet, & Simon, 2015; Hao & Chen, 2012; Jousselin et al., 2016; Overholt, Diaz, Rosskopf, Green, & Overholt, 2015). Therefore, HTS techniques are the first choice for studying the diversity of microbial communities (Degnan & Ochman, 2012).

In this study, the phylogenies of five species of mealybugs *Phenacoccus solenopsis* (Tinsley)*, Phenacoccus solani *(Ferris), *Dysmicoccus neobrevipes* (Beardsley), *Pseudococcus comstocki *(Kuwana), and *Planococcus minor* (Maskell) were studied base on the divergence of the mtCOI gene. Meanwhile, regions of the 16S rRNA genes of the P‐endosymbionts from the five mealybugs were sequenced to explore the phylogenetic congruence of the P‐endosymbionts and their hosts. In addition, to gain insight into the microbial abundance and diversity in mealybugs, the polymorphism of the bacteria present in the mealybugs was investigated using high‐throughput sequencing (HTS) techniques (Wang et al., 2018). The findings presented in this study will be helpful for future research on the coevolution of mealybugs and their endosymbionts.

## 2 MATERIALS AND METHODS

### 2.1 Insects and DNA extraction

The mealybug samples used in the study were collected from different areas. *Phenacoccus solenopsis* were collected from eggplant *Solanum melongena* in Linan, Zhejiang Province. *Pseudococcus comstocki *were collected from vineyard in Xianju, Zhejiang Province. *Dysmicoccus neobrevipes* were provided by Shanghai Customs and *Planococcus minor* were provided by Zhoushan Customs. Both of their host plants are pineapple *Ananas comosus*. *P. solani* was provided by Hangzhou Customs and its host plant is succulent plant *Senecio*. All mealybug samples were collected individually in separate microfuge tubes containing 75% ethanol and were stored at −20°C for further study. The out‐groups, *Icerya purchasi *(Maskell), were collected from *Citrus medica* in Xiamen, Fujian Province. *Asiacornococcus kaki *(Kuwana) was collected from *Diospyros* in Heze, Shandong Province. For mtCOI gene amplification and endosymbionts identification, an individual insect DNA (three replicates per species) was extracted. For microbiome analysis, the mixed five individuals were extracted (three replicates per species). All DNA was extracted using the E.Z.N.A.^®^ Insect DNA Kit (Omega Bio‐Tek, Norcross, GA, USA).

### 2.2 Genetic identification of Mealybugs samples

The mtCOI gene of insects (each species were repeated three times) was amplified using forward primer‐M2183 (5′‐CAACATTTATTTTTGATTTTTTGG‐3′) and reverse primer‐M2568 (5′‐GCWACWACRTAATAKGTATCATG‐3′) (Gullan et al., 2003). The gene was amplified with TaKaRa Taq™. The reaction conditions were 95°C for 5 min thereafter by 35 cycles of 95°C for 1 min, 52°C for 1 min and 72°C for 1.5 min with a final elongation step for 5 min at 72°C. The PCR products were sent to Sangon Biotech for sequencing (Shanghai, China).

### 2.3 Endosymbionts identification of Mealybugs samples

The 16S rRNA gene was amplified using the universal primers 16 s‐27F (5' AGAGTTTGATCMT GGCTCAG‐3') and 16 s‐1495R (5'‐CTACGGCTACCTTGTTACGA‐3') (Barzanti et al., 2007). The reaction conditions were five cycles of 95°C for 30 s, 55°C for 30 s, and 72°C for 2 min followed by 25 cycles of 95°C for 30 s, 50°C for 30 s, and 72°C for 2 min and 72°C 10 min. The PCR product was purified and then was cloned using pEASY‐T1 Cloning Kit according to the manufacturer's protocol. The sequencing process was performed by Sangon Biotech (Shanghai, China).

### 2.4 Molecular phylogenetic and evolutionary analyses

All sequences were aligned with Clustal X by MEGA (Version 6.06). The evolutionary divergence of different samples based on mtDNA CO1 used the Kimura 2‐parameter model. The phylogenetic trees were carried out by using the neighbor‐joining (NJ) method. Bootstrap values were calculated from 1,000 bootstrap replicates. The trees were rooted with a distinct out‐group. The genetic distance for these samples was calculated in the same software.

### 2.5 Microbiome identification of the mealybugs with distinct regions of 16S rRNA gene amplification and sequencing

The 16S ribosomal RNA (rRNA) V3‐V4 gene was amplified to assess the microbial diversity based on the Illumina HiSeq sequencing platform (Novogene Bioinformatics Technology Co., Ltd.). All amplicons with bright main strip were in the size range of 400–450 bp, and sequencing libraries were produced using TruSeq^®^ DNA PCR‐Free Sample Preparation Kit (Illumina, USA). After the library quality was assessed (only high‐quality sequences were remained), double‐ended sequencing was performed by using the method of paired‐end and paired‐end reads (250 bp) were produced. Paired‐end reads were merged using FLASH (Version 1.2.7) (Magoc & Salzberg 2011), and then, the newly synthesized sequences were retained as raw tags. In order to get the clean tags with the high quality, the QIIME (Version 1.7.0) quality controlled process was applied to quality filtering on the raw tags (Bokulich et al., 2013). Then, the tags were compared with Gold database using UCHIME algorithm (Edgar, Haas, Clemente, Quince, & Knight, 2011) to detect and removed chimera sequences. Finally, the obtained “Effective Tags” were for subsequent analysis (Haas et al., 2011).

### 2.6 Data analyses of HTS

#### 2.6.1 OTU cluster and species annotation

All Effective Tags of samples were clustered using Uparse software (Version 7.0.1001) (Edgar, 2013). The sequences with ≥97% identity were clustered into the same Operational Taxonomic Units (OTUs). At the same time, the highest frequency sequence of OTUs is selected as the representative sequence of OTUs according to its algorithm principle, which was conducted by Classifier algorithm (Wang, Garrity, Tiedje, & Cole, 2007). The MUSCLE (Version 3.8.31) software was used to perform rapid multiple sequence alignments to obtain the phylogenetic relationships of all OTUs representative sequences (Edgar, 2004). Finally, the data of each sample are homogenized, and then, the subsequent analysis was all based on the data after normalization. In order to further explore the phylogenetic relationships of different OTUs, and to compare the divergence of dominant species between different samples, we performed multiple sequence alignments. The phylogenetic trees were also carried out by neighbor‐joining (NJ) method using the programs MEGA (Version 6.06).

#### 2.6.2 Alpha diversity

The Estimated‐species (observed species), ACE, Chao1, Simpson, Shannon, and Goods‐coverage indices were calculated using the QIIME software and the dilution curve, rank abundance curve and species cumulative curve were plotted using the R software, which was applied to analyze the difference of alpha diversity index between the groups. A box‐plot chart of abundance estimators were also generated by the QIIME toolkit. One‐way ANOVA (SPSS.18.0) was implemented to evaluate the alpha diversity among groups.

#### 2.6.3 Beta diversity

Beta diversity analysis was service as an important tool to evaluate the distinction of all three samples in species complexity. The Unifrac distance was calculated using QIIME software to build the Unweighted Pair‐group Method with Arithmetic Means (UPGMA) clustering tree. Then, Principal component analysis (PCA) and Principal Coordinate Analysis (PCoA) graphs were carried out by using the R software. The LEfSe software was applied to analyze effect size, the default setting LDA score screening value of four. Two‐sided Student's *t* test was used for significance test of beta diversity difference between sample groups. Finally, to highlight differences in bacterial communities, the QIIME toolkit was chosen to construct a box‐plot of the dominant bacterial genus between different sample groups. And the composition of dominant bacterial genera among different groups was evaluated using the R statistical software (permutation test) at the level of Species (White, Nagarajan, & Pop, 2009).

## 3 RESULTS

### 3.1 Phylogenetic analysis of mealybugs and their endosymbionts

**Figure 1:**
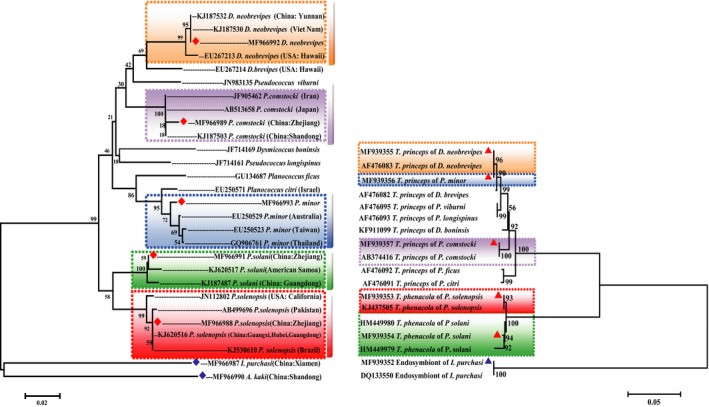
The phylogenetic tree based on mtDNA COI sequences divergence of the mealybugs and the divergence of the 16s rRNA sequences of their P‐endosymbionts. The phylogenetic trees were constructed by neighbor‐joining method using the programs MEGA (Version 6.06).Left, Relationship among mtDNA COI sequences of mealybugs and out‐group. The red diamond on behalf of five mealybugs (the orange dotted box region represents *Dysmicoccus neobrevipes*, the purple ones represent *P. comstocki*,the blue one represents *P. minor*,the red one represents *P. solenopsis*, and green one represents *P. solani*) sequenced in our study and the blue one represent the out‐groups sequenced in our study. Right, relationship of 16S rRNA sequences of their primary endosymbionts (the color of dotted line box are the same with the left). The red triangle on behalf of the P‐endosymbionts of five mealybugs sequenced in our study and the blue one represents out‐group sequenced in our study. The Information concerning the mealybugs used in this study and the accession numbers of the 16S rRNA of the P‐endosymbionts were shown in Supporting information Table S2

According to the phylogenetic tree, *P. solenopsis *was closely related to *P. solani*, sharing an identity of 95.03% (on average). *P. comstocki* shared a similarity of 94.38% (on average) with *D. brevipes* and 92.43% with *P. minor*. *D. neobrevipes* and *D. brevipes* were clustered in one clade. The results revealed that the intraspecies average genetic distances of the five mealybugs were 0.28% for *P. solenopsis*, 0.93% for *P. solani*, 0.35% for *P. comstocki*, 0.48% for *P. minor, and *0.5% for *D. neobrevipes* (Figure 1).

**Figure 2:**
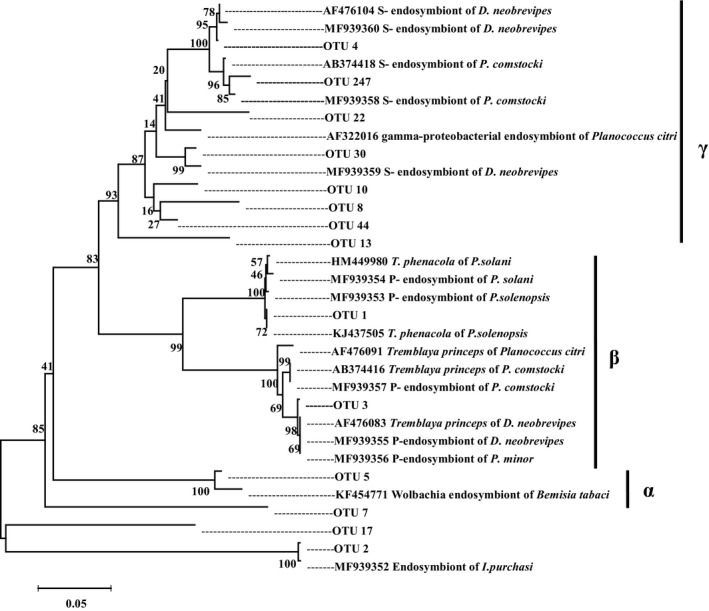
Dominant OTUs were inserted into a precompiled phylogenetic tree using Mega 6.06. The phylogenetic trees were also constructed by neighbor‐joining method using the programs MEGA. The P‐ endosymbionts and the S‐symbionts of five mealybugs (*Phenacoccus solenopsis*, *P. solani*, *P. comstocki*, *P. minor*, and *D. brevipes*) and out‐group (*Icerya purchasi*) shown in this figure was sequenced in our study and others were downloaded in NCBI. The information of OTUs was the same with that in Figure 3

Molecular phylogenetic analyses revealed that the β‐proteobacterial sequences of *P. comstocki*, *P. minor* and *D. brevipes *were placed within the clade of *T. princeps*. A fragment of approximately 1,500 bp within the 16S rRNA gene was sequenced in the endosymbionts, and these 16S rRNA sequences showed the highest sequence similarity (97.77%, on average) to that of *P. comstocki*, *P. minor,* and *D. brevipes* (Figure 1). Nevertheless, the P‐endosymbionts in *P. solenopsis *and *P. solani* were *T. phenacola*, which were 0.67% divergent from each other. Simultaneously, as shown in Figure 1, there was an 18.88% sequence divergence between *T. princeps* and *T. phenacola*. In addition, a fragment of approximately 1,500 bp within the 16S rRNA gene was also sequenced in the S‐symbiont in *P. comstocki* and *D. brevipes,* and the results are shown in Figure 2. Further information about the mealybugs used in this study and the accession numbers are listed in Supporting information Table S2.

### 3.2 Basic statistics of V3–V4 16S rRNA gene sequences by HTS

A total of 1,357,058 V3–V4 16S rRNA effective sequences reads were acquired from the total DNA extracts of seven species (three replicates, respectively), with an average of 64,622 valid sequences reads (the minimum of a sample was 57,895 sequences reads, and the maximum was 72,513 sequences reads). The average length of all valid sequences reads was 420 bp (Supporting information Table S3). The sparse curves of all samples showed that the database constructed by the target gene sequences had substantial abundance, and thus, sufficient depth in the analysis can be obtained from the data and information concerning the diversity of microbial community (Supporting information Figure S1).

**Figure 3:**
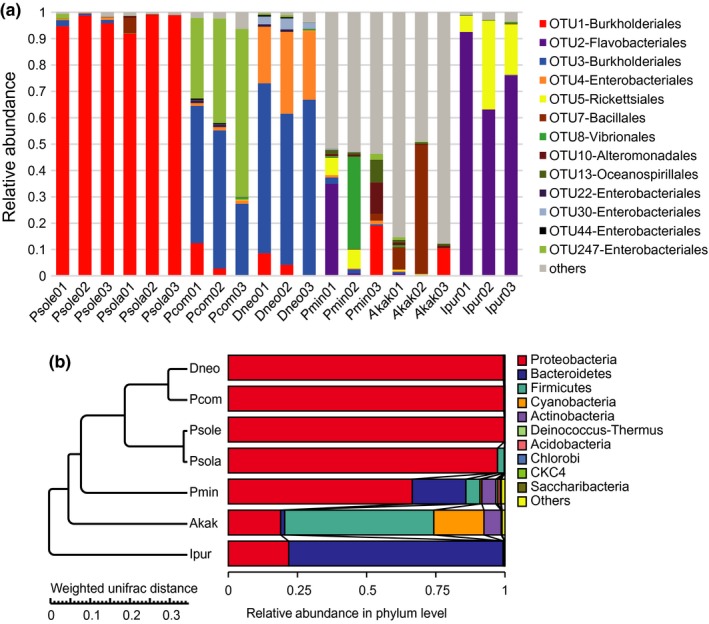
The relative abundances of enteric bacteria at the species (a) and phylum (b) level in five species mealybugs and two species out‐group was assessed using 16S rRNA high‐throughput sequencing. Microbial community composition for each microbiome analyzed. Dominant OTUs from each species. Only taxa with a relative abundance ≥1% in at least one sample were considered. Taxonomic classification includes genus when possible. OTUs from each dominant clade are colored with the same hue, and in all cases these dominant clades represent most of the total community

The bacterial diversity and relative abundance of all samples in the various taxonomies are presented in Supporting information Figure S2. Five phylogenetic groups, α‐, β‐, and γ‐proteobacteria, Flavobacteria, and Chloroplast, were identified as the major bacterial taxa of the scales bacterial community in all samples. The later probably came from stomach food. Based on relative abundance, the Proteobacteria (primarily β‐proteobacteria) was the most abundant bacterial phylum in the *Phenacoccus* populations, with an average relative abundance of over 93%. The γ‐proteobacteria had a higher relative abundance compared with other classes. The taxonomic distribution of each sample at the genus level is shown in Figure 3. The microbiome of *P. minor* was highly distinct from those of all other tested mealybugs, in which the dominant taxa were more abundant. The *P. comstocki *microbiome contained dominant taxa that were closely related to the dominant taxa in *D. brevipes*, while the microbiome of *P. solani *was similar to the dominant taxa in *P. solenopsis*. The bacteria from the Candidatus genus *T. princeps* (OTU3) were the most abundant bacteria in *P. comstocki *and *D. brevipes*, and the second most abundant bacteria were those them from Enterobacteriaceae family (OTU4, OTU30, and OTU247). Thus, these four OTUs represented between 83.81 and 95.28% of rRNA gene amplicon sequences from each sample. Similarly, 1–3 dominant OTUs within the *Phenacoccus *clade were detected, among which OTU1 was highly enriched in *Phenacoccus* (*P. solani* and *P. solenopsis*). In addition, all core bacteria in *P. minor*, such as OTU2, OTU5, OTU8, OTU10, OTU13, and others, found in our study were rarely detected in the *Phenacoccus* populations (Figure 3a). Moreover, the bacteria *Wolbachia* (OTU5) were detected in *P. minor*, with an average relative abundance of >4%.

### 3.3 Characterization of the microbiome of mealybugs

To assess the bacterial community structure succession, the corresponding results revealed an apparent trend in the relative abundance of distinct bacterial taxa (Figure 3b). In five mealybugs, the Proteobacteria was the dominant phylum, whereas in the two out‐groups, Bacteroidetes and Firmicutes were the dominant phyla (Figure 3).

**Figure 4:**
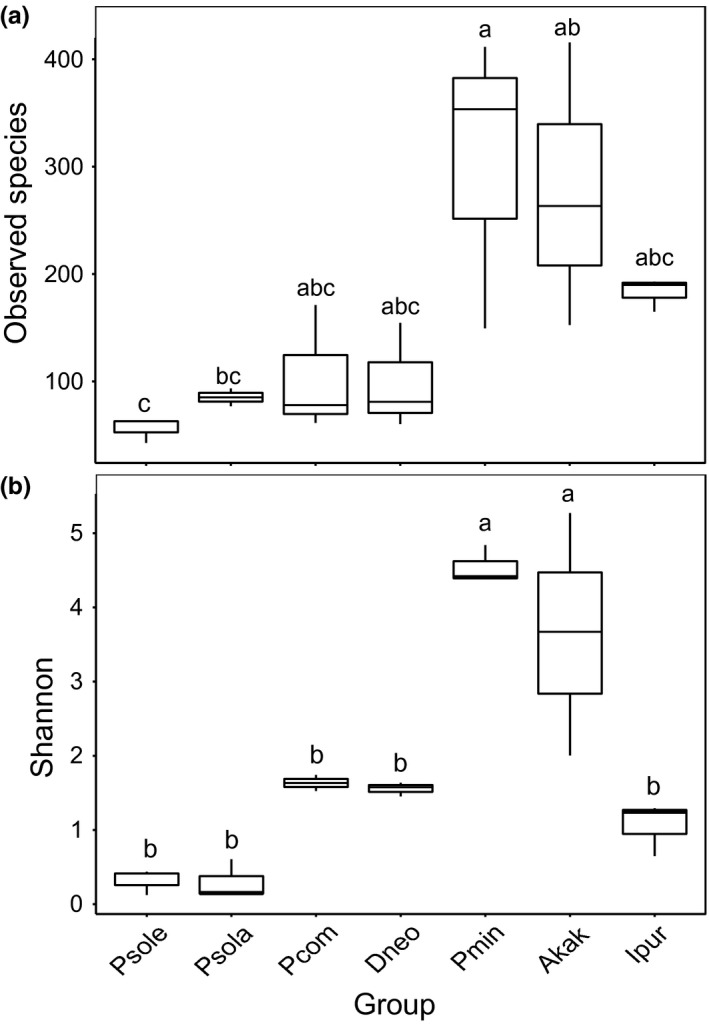
Differences in bacterial community diversity, richness and structure among seven samples. A box‐plot of richness estimators observed species (a) and Shannon diversity index (b) were examined by 16S high‐throughput sequencing was constructed using the QIIME toolkit. Alpha diversities were further tested by comparing the alpha diversity indexes between groups using one‐way ANOVA (SPSS. 18.0), *n* = 3 per group. Statistically significant differences are indicated. For observed species comparison, *Planococcus minor* and *Asiacornococcus kaki* had significantly higher number than others (*p* = 0.007, *F* = 4.786, Figure 4a). We also found significant difference in Shannon diversity between *P. minor* and other four mealybugs (*p* = 0.000, *F* = 18.729, Figure 4b). The top and bottom boundaries of each box indicate the 75th and 25th quartile values, respectively, and lines within each box represent the 50th quartile (median) values

**Figure 5:**
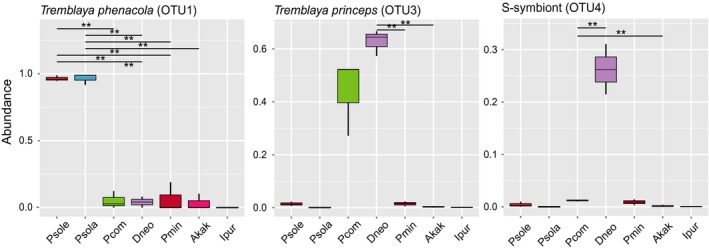
The relative abundances of P‐ and S‐ bacteria at the phylum, genus and species level in the five species mealybugs and two out‐groups species. Metalats analysis using the R software (Version 2.15.3) at the classification level, make the permutation test between groups, get p value, and then use Benjamini and Hochberg False Discovery Rate method for correcting the value of p, q value obtained. Finally, according to the q value marked the significance, to find significant differences in symbiotic bacteria (**q < 0.01)

The box‐plot of the dominant bacterial genera constructed among the different sample groups (Figures 4 and 5) indicated that the bacterial community compositions were further confirmed by the clear clustering of the dominant bacterial genus and species corresponding to different populations in the heat map, as shown in Supporting information Figures S3 and S4. Three alpha diversity measurements were calculated, including the Shannon diversity index, observed species (observed OTUs), and Chao1 (Estimated Richness) (Table 1). For observed species comparison, *P. minor* and *E. kaki* had a significantly higher number of observed and estimated (Chao1) OTUs compared with the other species (*p* = 0.007, *F* = 4.786, Figure 4a). We also found significant differences between *P. minor* and the other four mealybugs (*p* = 0.000, *F* = 18.729, Figure 4b) in Shannon diversity. Moreover, the two indicators of *P. comstocki* and *D. brevipes* were significantly higher than that of *P. solenopsis*. Meanwhile, no significant differences in richness were observed between *P. solenopsis *and *P. solani*.

**Table 1:** HTS Sequencing summary of microbiomes from mealybugs

Sample ID	Sequences Retrieved	Number of OTUs	Estimated Richness	Shannon	Num OTU > 1% Abundance	GC content
Psole01	66,640	62	69.814	0.438	3	49.90
Psole02	64,341	42	50.141	0.124	1	49.72
Psole03	61,525	61	68.501	0.390	2	49.85
Psola01	70,960	91	106.853	0.606	2	49.68
Psola02	59,937	83	98.810	0.134	1	49.49
Psola03	61,027	75	85.041	0.150	1	49.52
Pcom01	66,907	76	91.430	1.743	7	52.94
Pcom02	68,025	60	78.713	1.525	6	52.97
Pcom03	58,235	166	190.675	1.634	5	51.65
Dneo01	72,513	79	95.985	1.576	4	53.90
Dneo02	66,011	59	65.401	1.637	4	53.58
Dneo03	57,895	150	218.779	1.452	3	54.05
Pmin01	62,919	342	360.022	4.379	13	51.22
Pmin02	63,763	398	422.432	4.407	9	51.13
Pmin03	64,609	145	152.072	4.840	15	53.67
Ekak01	59,155	402	424.079	5.273	11	54.02
Ekak02	61,178	255	264.810	3.671	13	53.14
Ekak03	68,386	148	164.381	2.004	7	53.14
Ipur01	69,644	187	224.176	0.648	2	45.67
Ipur02	72,743	185	242.068	1.294	2	46.44
Ipur03	60,645	160	194.825	1.247	2	46.14

Although similar microbial richness was observed between mealybugs, the community structures were significantly different among the microbiomes of all tested organisms, of which *P. solani* and *P. solenopsis *shared a high similarity. Additionally, the results confirmed that two species of mealybugs had low overall species richness with 42–91 detected OTUs and housed 1–3 dominant taxa (Table 1). As a result, only 1–3 OTUs had >1% abundance in populations from *P. solani* and *P. solenopsis*, while 4–5 OTUs with >1% abundance were found in other mealybugs (Table 1). The Shannon diversity indices ranged from 0.124 to 0.606 in *P. solani* and *P. solenopsis*, and the microbial communities sampled from *P. minor*, *P. comstocki*, and *D. brevipes *were significantly more diverse than those from *P. solani* and *P. solenopsis* (Table 1). The Estimated Richness and diversity in *E. kaki* were higher compared with the others. According to the GC content, *P. solani* and *P. solenopsis* belong to *Phenacoccus*, with a GC content of approximately 50%, whereas the GC content of the others species was slightly higher (except the out‐group *I. purchasi*, with an average GC content of 46%). The OTU number and alpha diversity metrics for *P. minor* were higher compared to those from the other four mealybugs (Table 1).

A Venn diagram was used to compare the similarities and differences between the communities in the samples. The *P. solenopsis, P. solani,* *P. comstocki*, *D. brevipes*, and *P. minor* communities had 40 OTUs in common (Supporting information Figure S5A), with unique OTUs totaling 12, 46, 47, 33, and 330 in the *P. solenopsis, P. solani,* *P. comstocki*, *D. brevipes*, and *P. minor *communities, respectively (Figure S5B).

### 3.4 Clustering patterns of different species of scales

**Figure 6:**
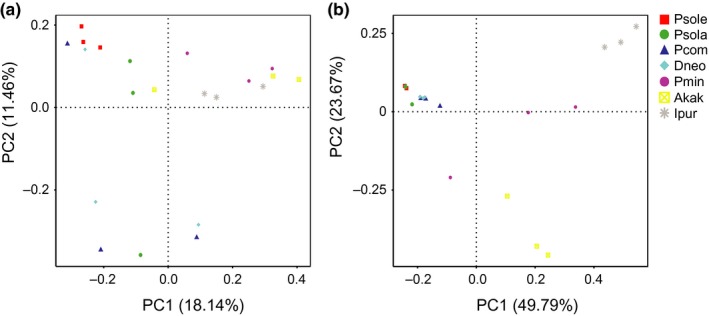
Two‐dimensional principal coordinates analysis (PCoA) plot of unweighted (abundance is ignored) (a) and weighted (abundance is considered) (b) unifrac distance matrices for seven samples

According to the unweighted unifrac PCoA, the samples were all clustered into three distinct clusters (Figure 6a). On the basis of principal coordinate 1 (PC1) and PC2 analyses (18.14% and 11.46% of variance explained, respectively), the microbial communities of *P. minor* were separated from the microbial communities of the four other mealybugs and clustered into the clades of out‐groups. The separation between samples across different species and the similarity between four mealybugs (*P. solani*, *P. solenopsis*, *P. comstocki,* and *D. brevipes*) were more notable on the weighted unifrac‐based plot. According to the weighted unifrac PCoA, all of the samples were grouped into two distinct clusters based on PC1 and PC2 analyses (49.79% and 23.67% of variance explained, respectively; Figure 6b). Four species of mealybugs formed a unique cluster, separate from the other samples. However, as shown in Figure 6, sample P.min 3 clustered separately from samples in the same group and showed a different pattern of relative abundance (this might have been caused by sampling error or poor sample quality).

## 4 DISCUSSIONS

### 4.1 Phylogenetic analysis of mealybugs and their endosymbionts

The phylogenetic tree showed that the β‐proteobacterial sequences from *P. comstocki*, *P. minor,* and *D. brevipes *were placed within the clade of *T. princeps*, while the P‐endosymbionts in *P. solenopsis *and *P. solani* were identified as *T. phenacola*. A number of mealybugs harbor secondary γ‐proteobacterial endosymbionts, which are contained within the cell of the β‐proteobacteria *T. princeps *(Von Dohlen et al., 2001). The unusual nested relationship between the β‐proteobacteria and γ‐proteobacteria has been well studied in recent research (Gatehouse et al., 2011; Kono et al., 2008; Sergio et al., 2013). According to previous studies, mealybugs, especially the mealybugs, display a unique nested model where in the β‐proteobacteria *T. princeps* contains secondary bacteria (Gatehouse et al., 2012; Von Dohlen et al., 2001). For example, *T. princeps* in *Planococcus citri* harbors a γ‐proteobacterium, “*Candidatus* Moranella endobia.” Similar to *P. citri*, the results showed that the primary endosymbionts of *P. comstocki*, *P. minor*, and *D. brevipes* were the β‐proteobacteria *T. princeps*. In addition, we also detected two distinct secondary bacteria in *P. comstocki* and *D. brevipes* by sequencing the 16S rRNA gene. Based on the phylogenetic tree analysis, we can speculate that the secondary bacteria of *P. comstocki* and *D. brevipes* were probably also present in β‐proteobacteria, suggesting that these two mealybugs also have the same nested structure.

From many previous studies, we found that various insects, such as the whitefly, mealybugs, and others, exhibited distinct but stable symbiont systems and a close‐knit host–symbiont connection over evolutionary time, implying that the development of symbiotic systems generally mirror the phylogenetic relationships of the host insects (Clark et al., 2000; Thao et al., 2000; Thao & Baumann, 2004; Rao et al., 2015). This phenomenon is similar to the findings on “*Ca*. Tremblaya” in mealybugs in this study. The horizontal transfer of genes influences the endosymbionts of insects in the Phenacoccinae, which clearly reveal the mealybugs’ two typical endosymbiotic systems of historical evolution (Lopez‐Madrigal et al., 2014). Interestingly, the previous study also revealed that the theory of host–symbiont symbiosis can also be applied to gut symbionts. The stable relationship between host and symbiont implies the significant biological role of the symbiont in the host insects, which is preserved by generations in a vertical transfer manner. The findings of Kikuchi and Hosokawa strongly confirmed that the intracellular or extracellular environment had little effect on symbiont genomic evolutionary change (Kikuchi et al., 2009). As we expected, our results are consistent with the assumption of co‐diversification of “*Ca*. Tremblaya” and the host mealybug.

### 4.2 Basic statistics of V3–V4 16S rRNA gene sequences by HST

Interestingly, *P. comstocki *and *D. neobrevipes* harbor a distinct dominant Enterobacteriaceae group. The OTU4 and OTU247, which belong to Enterobacteriaceae, were abundant in *P. comstocki *and *D. neobrevipes*. OTU4 was closer to γ‐proteobacteria in other mealybugs, which means that OTU4 was nested in β‐proteobacteria (Figure 3). However, this pattern is different from the nested endosymbiotic pattern of *P. comstocki *and *D. neobrevipes,* consisting primarily of the β‐proteobacterial *T. princeps* that contains the γ‐proteobacterium (Figure 3). The abundance of OTU4 in *D*. *neobrevipes* was higher than in other species (Figure 5). Whether the common origin of *T. princeps* and *T. phenacola* had already been connected with the γ‐proteobacterium or not might have influenced their genome structure. The bacteria *Staphylococcus *(OTU7) and *Bacillus *(OTU6) are common members of the gut microbial communities in insects such as whitefly *Bemisia tabaci* and *Apriona germari*, and these bacteria have been reported for their role in reducing the pathogenic gut microbe population by maintaining the gut pH (Takatsuka & Kunimi, 2000). Moreover, the association with *Bacillus*, *Staphylococcus,* and *Micrococcus* at all *B. tabaci* developmental stages indicates a symbiosis and, indeed, suggests the detoxification of toxic substances (Genta, Dillon, Terra, & Ferreira, 2006; Lauzon, Potter, & Prokopy, 2003). Previous studies have reported the role of bacterial symbionts in insecticide resistance, confirming that bacterial symbionts of the genus *Burkholderia* in insects could degrade fenitrothion and thus enhance insect resistance to the insecticide. At the same time, we speculated that the insecticide‐degrading symbiotic bacteria could transfer horizontally through different pests of the same species (Kikuchi et al., 2009). The mealybugs in this study also contain endosymbionts of the genus *Burkholderia,* and some of them harbor *Staphylococcus *(OTU7) and *Bacillus* (OTU6), which may play a role in conferring insecticide resistance to mealybugs. These symbiotic bacteria may be obtained by insects from environmental soil or plants (Li et al., 2017). Vibrionaceae (OTU8) is a family of γ‐proteobacteria, which is typically found as symbionts in deep‐sea creatures and was also abundant in *P. minor *(average relative abundance was approximately 11.90%) in our study. Certain members of the genus *Vibrio* cause diarrhea or gastroenteritis in humans (Lin, Kumagai, Baba, Mekalanos, & Nishibuchi, 1993). Other members of the genus may cause a rapidly progressive hemorrhagic septicemia that can account for high mortalities among marine animals (Singer, Choe, Schmidt, & Makula, 1992). At the same time, this symbiont can also generate neurotoxins, such as tetrodotoxin (Simidu, Noguchi, Hwang, Shida, & Hashimoto, 1987), which protect itself from the predation of its natural enemies (Johnson et al., 2018).

### 4.3 Characterization of the microbiome of mealybugs

Alpha diversity was applied in analyzing the complexity of species diversity, and a Beta diversity analysis was used to assess discrepancies of samples in species integrality. Through these analyses, we observed that *P. minor *harbors a rich microbiome. The OTU number and alpha diversity metrics for *P. minor* were higher compared with the samples collected from the other four mealybugs. Moreover, the GC content data are also listed in Table 1. The measurement of GC content has an intimate correlation with the amino acid content of proteins, codon usage in messenger ribonucleic acid, and other properties of biology fields. The greatest potential of GC analysis may be its usefulness as a marker for classification that can be used to differentiate microorganisms with similar phenotypes (Mesbah, Premachandran, & Whitman, 1989). Previous research showed that the 16S rRNA gene sequences of *T. princeps *were GC‐rich (39%–46% AT) in comparison with those of free‐living β‐proteobacteria (43%–47% AT) (Baumann, Thao, Hess, Johnson, & Baumann, 2002; Mccutcheon & Dohlen, 2011). By contrast, the 16S rRNA gene sequences of *T. phenacola* were AT‐rich (49%–54% AT) (Gruwell et al., 2010). This study showed that the GC content of *P. solani* and *P. solenopsis *is approximately 50%, but that of *P. minor*, *P. comstocki* and *D. neobrevipes* are higher (Table 1). For amino acid synthesis, *T. princeps* differed from the other endosymbionts in that there were more amino acid‐encoding regions with a high GC content compared to the P‐endosymbiont in aphid and P‐endosymbiont in phylloxera (Clark, Baumann, Thao, Moran, & Baumann, 2001). These results correlated with the GC contents of the DNAs, which encode these proteins (Baumann et al., 2002). However, so far, there have been few studies on the relationship between inducing pathogens and insect invasion potentials, especially in the case of phytophagous insects, including invasive insects, which contain large amounts of symbiotic bacteria (Douglas, 2009). Some studies have proposed that there is a connection between the intracellular lifestyle of eukaryotic symbionts and pathogens with a low GC content. Interestingly, the bacterial communities associated with the living host environment are essential factors shaping the microbiota of insects (Linnenbrink et al., 2013). As a result, we concluded that certain groups within the microbiota and the bacterial richness in the insect gut may be affected by the host diet, habitat, and developmental stage (Yun et al., 2014). Certainly, the diversity of plant nutrient sources may also affect the abundance of bacteria in the host insects that feed on plants (Francis & Currie, 2003; Parkinson, Gobin, & Hughes, 2016).

### 4.4 Clustering patterns of different species of scales

*Phenacoccus solenopsis* is an invasive pest in a wide array of host plants, which was first reported to be found in Guangzhou, China, and has spread to other provinces since then (Wu et al., 2015). In the study, all of the mtCOI data from the *P. solenopsis* samples from China had a closer distant phylogenetic relationship with those from Pakistan and other Asian countries (Figure 1). These experimental results are consistent with previously reported results (Wu et al., 2015; Zhe et al., 2013). A geographical population analysis was conducted on another four kinds of mealybugs. The results of that study were similar to those of the present study, in that *D. neobrevipes* had a close relationship with the species from Vietnam. To adapt to new conditions, mealybugs may face changes that may be inherited by invasive species (Wu et al., 2015). Therefore, mealybugs of different geographical populations have various adaptations to different environments and thus affect endosymbionts indirectly (Ross, Shuker, Normark, & Pen, 2012). In this study, we demonstrated that the *P. solani* and *P. solenopsis* have a simple endosymbiotic system involving *T. phenacola,* which belongs to the β‐proteobacteria, contained within phenacoccinae mealybugs. The stable host–symbiont association is implicit in important biological roles of the endosymbionts for the host insect and rigorous vertical transmission of the symbiont through host generations. In addition, the results of HTS revealed that the observed species and Shannon diversity indices of *P. solenopsis *were lower than those of three native species (*P. comstocki, E. kaki *and *I. purchasi*) and two exotic species (*D. brevipes *and *P. minor*). Meanwhile, no significant differences in richness were observed between *P. solenopsis *and *P. solani*. Thus, the coexistence of endosymbionts may contribute to the invasiveness of exogenous harmful insects, which may be due to the presence of a single P‐endosymbiont and less abundant S‐symbionts in *P. solenopsis*. Genetic reductions in invasive species have been widely documented. For example, the loss of endosymbiotic bacteria in invasive populations is common in ant species (Rey et al., 2013; Yang et al., 2010). Another study found that the geographic invasion of mosquitoes is related to their symbiotic microbiome communities. Invasive species have a significantly reduced diversity of microbial populations in their hosts compared to native species (Minard et al., 2015). An important reason for the reduction in the diversity of endosymbiotic bacteria in the invasive species may be that the invasive species can gradually replace the native species. In future studies, it will be valuable to collect more samples of mealybugs from different parts of China and analyze the genetic structure of mealybugs using different and diverse molecular markers. This will help us to further understand the sources of invasive mealybugs and the relation between their invasion and endosymbiotic bacteria.

## CONFLICT OF INTERESTS

The author(s) declare that they have no competing interests.

## AUTHOR CONTRIBUTIONS

QR and HMW conceived and designed the experiments. DL, LZ, WDS, and XLL performed experiments. LD, QR, and XYL analyzed the data. LD and QR drafted the manuscript. All authors read and approved the final manuscript.

## Data Availability

Novel nucleotide sequences have been deposited in the GenBank nucleotide database (Accession nos.: partial mtCOI sequences of mealybugs, MF966987‐MF966993; 16S rRNA sequences of primary endosymbionts, MF939352‐MF939360). All other data are presented in the Supporting information.
