# Supplementary material for: Phylogenetic analyses and characteristics of the microbiomes from five mealybugs (Hemiptera: Pseudococcidae)
Source: Ecol Evol. 2019 Jan 21;9(4):1972–84. doi: 10.1002/ece3.4889 (PMC6392364; doi:10.1002/ece3.4889)
Supplement: Supplementary file 1 [file ECE3-9-1972-s001.pdf]

Table S1: The information about the mealybugs used in this study and the accession numbers of the 16S rRNA of the P-endosymbionts

Mealybug species	COI	16S rRNA (P-endosymbiont)
Phenacoccus solenopsis	MF966988ZA*	MF939353*
	JN112802（USA）	KJ437505
	AB499696（Pakistan）	
	KJ620516（China）	
	KJ530610（Brazil）	
Phenacoccus solani	MF966991*	MF939354*
	KJ187487 (China: Guangdong)	HM449979
	KJ620517(American Samoa)	
Phenacoccus peruvianus	JF714164	KF444174
Phenacoccus madeirensis	JF714167	KF444180
Dysmicoccus neobrevipes	MF966992*	MF939355*
	GQ906757（Philippines）	AF476083
	KJ187530 (Viet Nam)	
Dysmicoccus brevipes	EU267214	AF476082
Pseudococcus comstocki	MF966989*	MF939357*
	JF905462(Iran)	AB374416
	AB513658 (Japan)	
	KJ187503 (China: Shandong)	
Pseudococcus longispinus	AB512118	AF476093
Pseudococcus viburni	GU134685	AF476095
Planococcus ficus	GU134687	AF476092
Planococcus citri	EU250571	AF476091
Planococcus minor	MF966993*	MF939356*
	EU250529(Australia: Queensland)	-
	GQ906761(Thailand)	
	EU250529 (Australia）	
Icerya purchasi	MF966987*	MF939352*、DQ133550
Asiacornococcus kaki	MF966990*	—

***in this study**

Table S2: OUT's data. Data was obtained using the Illumina Miseq2500-pyrosequencing

Sample Name	Raw PE	Combined	Qualified	Base(nt)	AvgLen(nt)	GC%	Effective%
**Psole01**	90,231	79,190	66,640	28,629,218	431	49.90	73.70
**Psole02**	84,377	75,659	64,341	27,702,373	431	49.72	76.23
**Psole03**	80,842	72,143	61,525	26,448,604	431	49.85	75.97
**Psola01**	95,394	84,317	70,960	30,436,627	430	49.68	74.12
**Psola02**	80,981	71,040	59,937	25,783,839	431	49.49	73.93
**Psola03**	80,998	77,953	61,027	26,242,662	431	49.52	75.20
**Pcom01**	89,372	79,225	66,907	28,577,218	429	52.94	74.54
**Pcom02**	88,894	79,980	68,025	29,087,770	429	52.97	76.32
**Pcom03**	91,309	85,312	58,235	24,645,559	428	51.65	63.02
**Dneo01**	94,234	84,816	72,513	31,007,711	429	53.90	76.73
**Dneo02**	83,491	76,235	66,011	28,198,860	429	53.58	78.78
**Dneo03**	91,590	85,570	57,895	24,547,821	429	54.05	62.54
**Pmin01**	85,755	80,294	62,919	25,315,123	417	51.22	70.85
**Pmin02**	90,907	85,085	63,763	24,969,301	421	51.13	65.26
**Pmin03**	86,170	75,566	64,609	25,170,741	425	53.67	68.69
**Akak01**	82,028	78,725	59,155	23,511,736	415	54.02	69.00
**Akak02**	87,552	83,722	61,178	24,310,419	422	53.14	65.86
**Akak03**	88,173	80,237	68,386	28,752,318	428	53.14	76.10
**Ipur01**	93,554	89,859	69,644	29,224,371	423	45.67	73.91
**Ipur02**	95,770	91,910	72,743	30,060,512	417	46.44	75.30
**Ipur03**	81,300	78,256	60,645	25,173,217	420	46.14	73.76

Table S3: Richness estimator of 21 samples

Sample name	Observed species	shannon	simpson	chao1	ACE	Goods coverage	PD-whole tree
**Psole01**	62	0.438	0.103	68.600	69.814	1	13.665
**Psole02**	42	0.124	0.024	46.091	50.141	1	14.615
**Psole03**	61	0.390	0.081	68.333	68.501	1	18.666
**Psola01**	91	0.606	0.158	103.667	106.853	1	14.500
**Psola02**	83	0.134	0.020	94.053	98.810	1	19.563
**Psola03**	75	0.15	0.023	83.571	85.041	1	9.453
**Pcom01**	76	1.743	0.619	86.688	91.430	1	8.528
**Pcom02**	60	1.525	0.567	79.000	78.713	1	12.830
**Pcom03**	166	1.634	0.519	184.700	190.675	0.999	15.404
**Dneo01**	79	1.576	0.531	85.577	95.985	1	13.778
**Dneo02**	59	1.637	0.571	63.091	65.401	1	21.061
**Dneo03**	150	1.452	0.486	203.036	218.779	0.999	18.715
**Pmin01**	342	4.379	0.854	361.091	360.022	0.999	60.261
**Pmin02**	398	4.407	0.850	425.973	422.432	0.999	70.829
**Pmin03**	145	4.840	0.926	148.750	152.072	1	28.930
**Akak01**	402	5.273	0.929	420.140	424.079	0.999	81.453
**Akak02**	255	3.671	0.746	265.500	264.810	1	43.402
**Akak03**	148	2.004	0.506	158.500	164.381	1	21.724
**Ipur01**	187	0.648	0.143	216.216	224.176	0.999	24.820
**Ipur02**	185	1.294	0.488	231.243	242.068	0.999	25.479
**Ipur03**	160	1.247	0.387	180.676	194.825	0.999	37.471

Figure S1

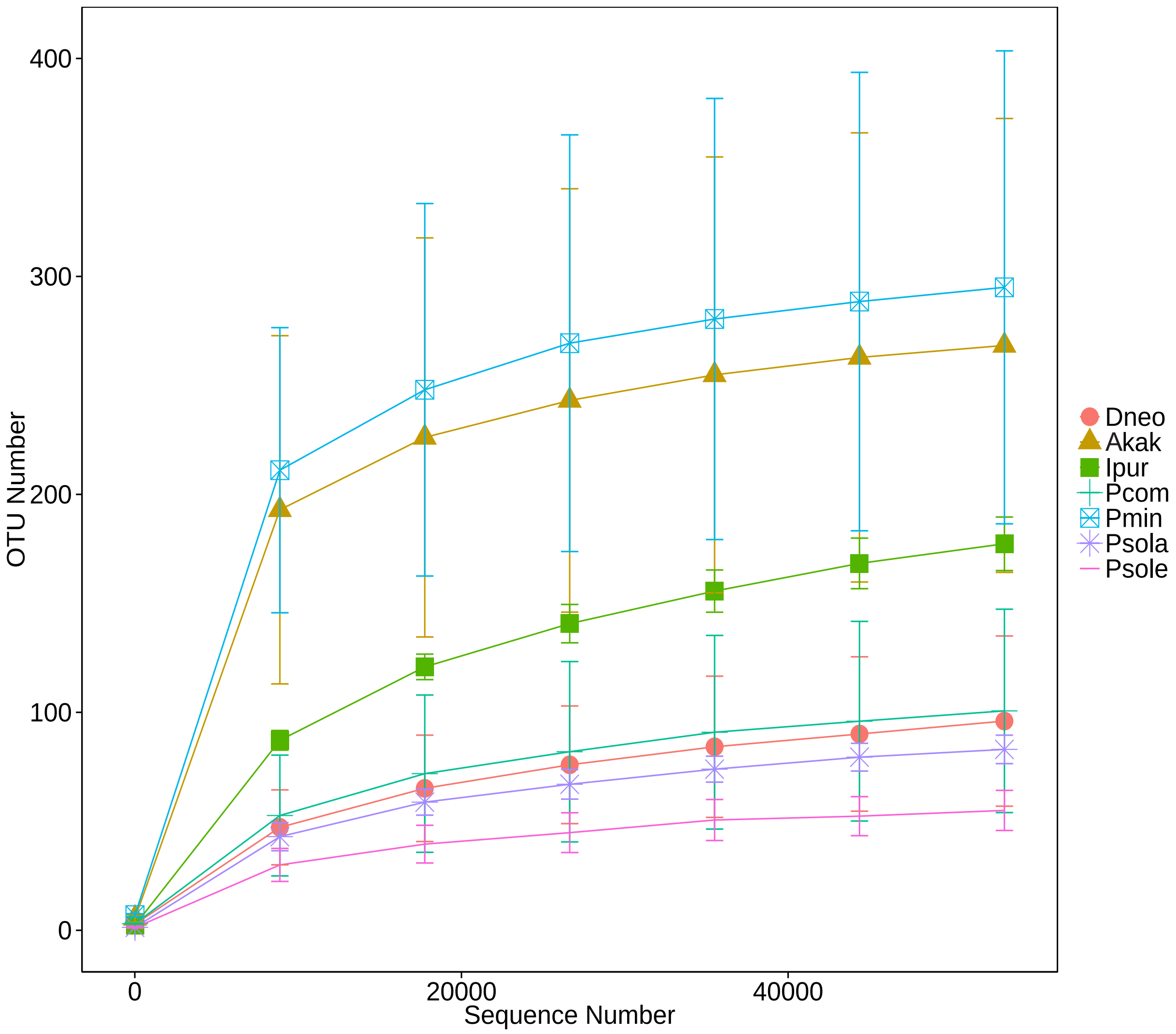

OTU Number
400
300
200
100
0
0
20000
40000
Sequence Number
Dneo
Akak
Ipur
Pcom
Pmin
Psola
Psole

Figure S2

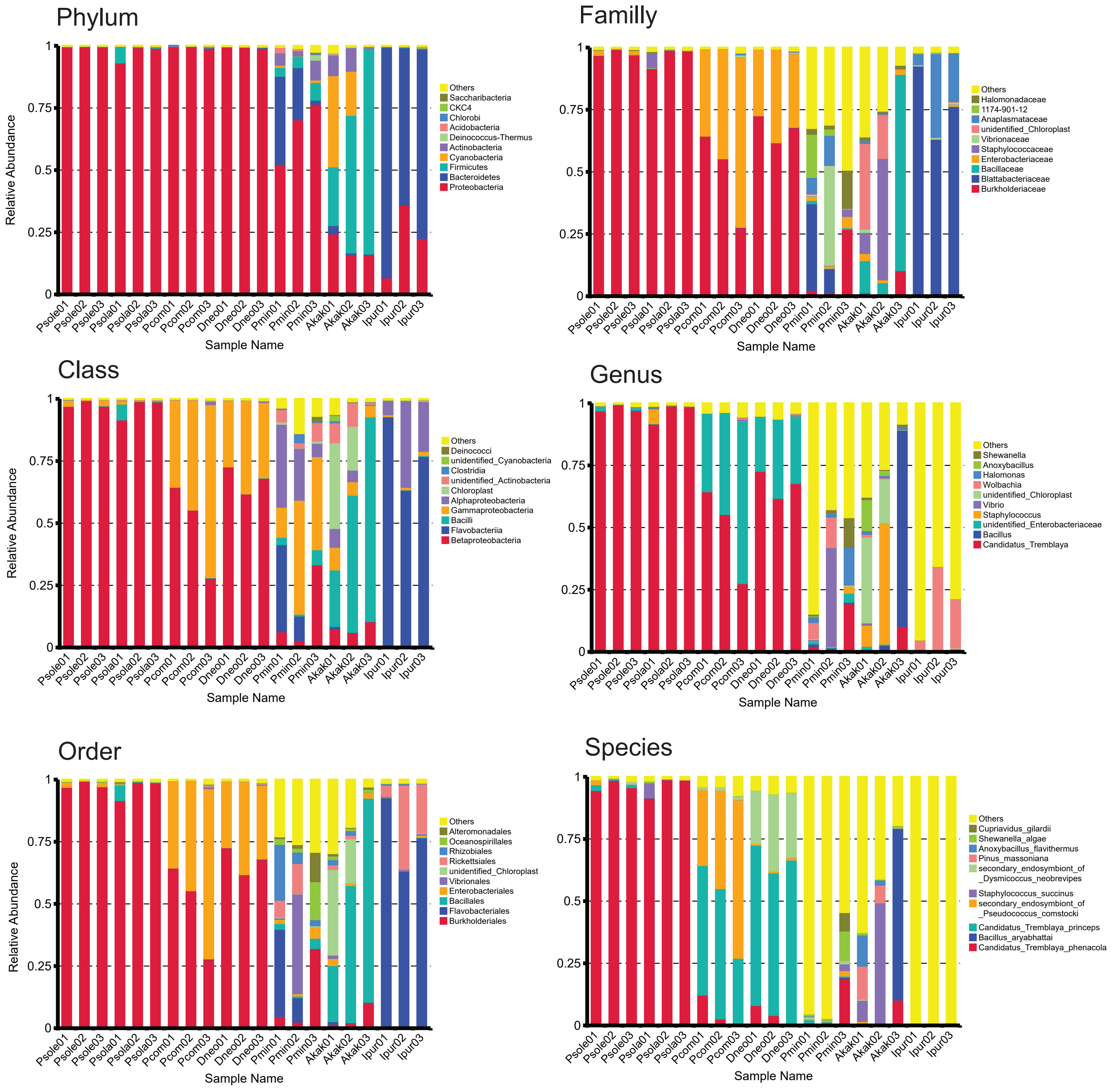
Phylum
Relative Abundance
Sample Name
Psole01 Psole02 Psole03 Psola01 Psola02 Psola03 Pcom01 Pcom02 Pcom03 Dneo01 Dneo02 Dneo03 Pmin01 Pmin02 Pmin03 Akak01 Akak02 Akak03 Ipur01 Ipur02 Ipur03
Others
Saccharibacteria
CKC4
Chlorobi
Acidobacteria
Deinococcus-Thermus
Actinobacteria
Cyanobacteria
Firmicutes
Bacteroidetes
Proteobacteria
Familly
Others
Halomonadaceae
1174-901-12
Anaplasmataceae
unidentified_Chloroplast
Vibrionaceae
Staphylococcaceae
Enterobacteriaceae
Bacillaceae
Blattabacteriaceae
Burkholderiaceae
Class
Others
Deinococci
unidentified_Cyanobacteria
Clostridia
unidentified_Actinobacteria
Chloroplast
Alphaproteobacteria
Gammaproteobacteria
Bacilli
Flavobacteriia
Betaproteobacteria
Genus
Others
Shewanella
Anoxybacillus
Halomonas
Wolbachia
unidentified_Chloroplast
Vibrio
Staphylococcus
unidentified_Enterobacteriaceae
Bacillus
Candidatus_Tremblaya
Order
Others
Alteromonadales
Oceanospirillales
Rhizobiales
Rickettsiales
unidentified_Chloroplast
Vibrionales
Enterobacteriales
Bacillales
Flavobacteriales
Burkholderiales
Species
Others
Cupriavidus_gilardii
Shewanella_algae
Anoxybacillus_flavithermus
Pinus_massoniana
secondary_endosymbiont_of _Dysmicoccus_neobrevipes
Staphylococcus_succinus
secondary_endosymbiont_of _Pseudococcus_comstocki
Candidatus_Tremblaya_princeps
Bacillus_aryabhattai
Candidatus_Tremblaya_phenacola

Figure S3

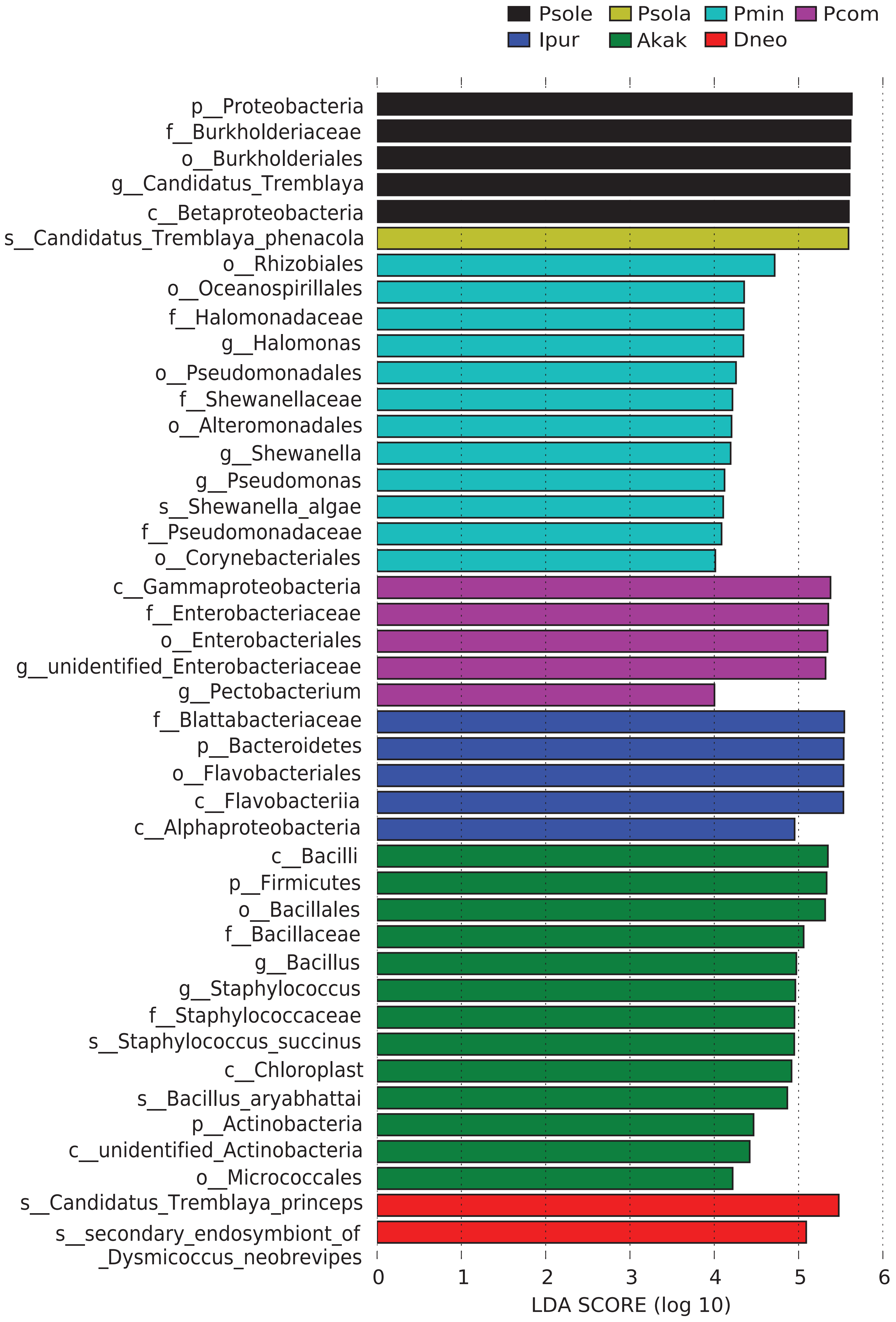
Psole
Psola
Pmin
Pcom
Ipur
Akak
Dneo
p__Proteobacteria
f__Burkholderiaceae
o__Burkholderiales
g__Candidatus_Tremblaya
c__Betaproteobacteria
s__Candidatus_Tremblaya_phenacola
o__Rhizobiales
o__Oceanospirillales
f__Halomonadaceae
g__Halomonas
o__Pseudomonadales
f__Shewanellaceae
o__Alteromonadales
g__Shewanella
g__Pseudomonas
s__Shewanella_algae
f__Pseudomonadaceae
o__Corynebacteriales
c__Gammaproteobacteria
f__Enterobacteriaceae
o__Enterobacteriales
g__unidentified_Enterobacteriaceae
g__Pectobacterium
f__Blattabacteriaceae
p__Bacteroidetes
o__Flavobacteriales
c__Flavobacteriia
c__Alphaproteobacteria
c__Bacilli
p__Firmicutes
o__Bacillales
f__Bacillaceae
g__Bacillus
g__Staphylococcus
f__Staphylococcaceae
s__Staphylococcus_succinus
c__Chloroplast
s__Bacillus_aryabhattai
p__Actinobacteria
c__unidentified_Actinobacteria
o__Micrococcales
s__Candidatus_Tremblaya_princeps
s__secondary_endosymbiont_of
_Dysmicoccus_neobrevipes
0
1
2
3
4
5
6
LDA SCORE (log 10)

Figure S4

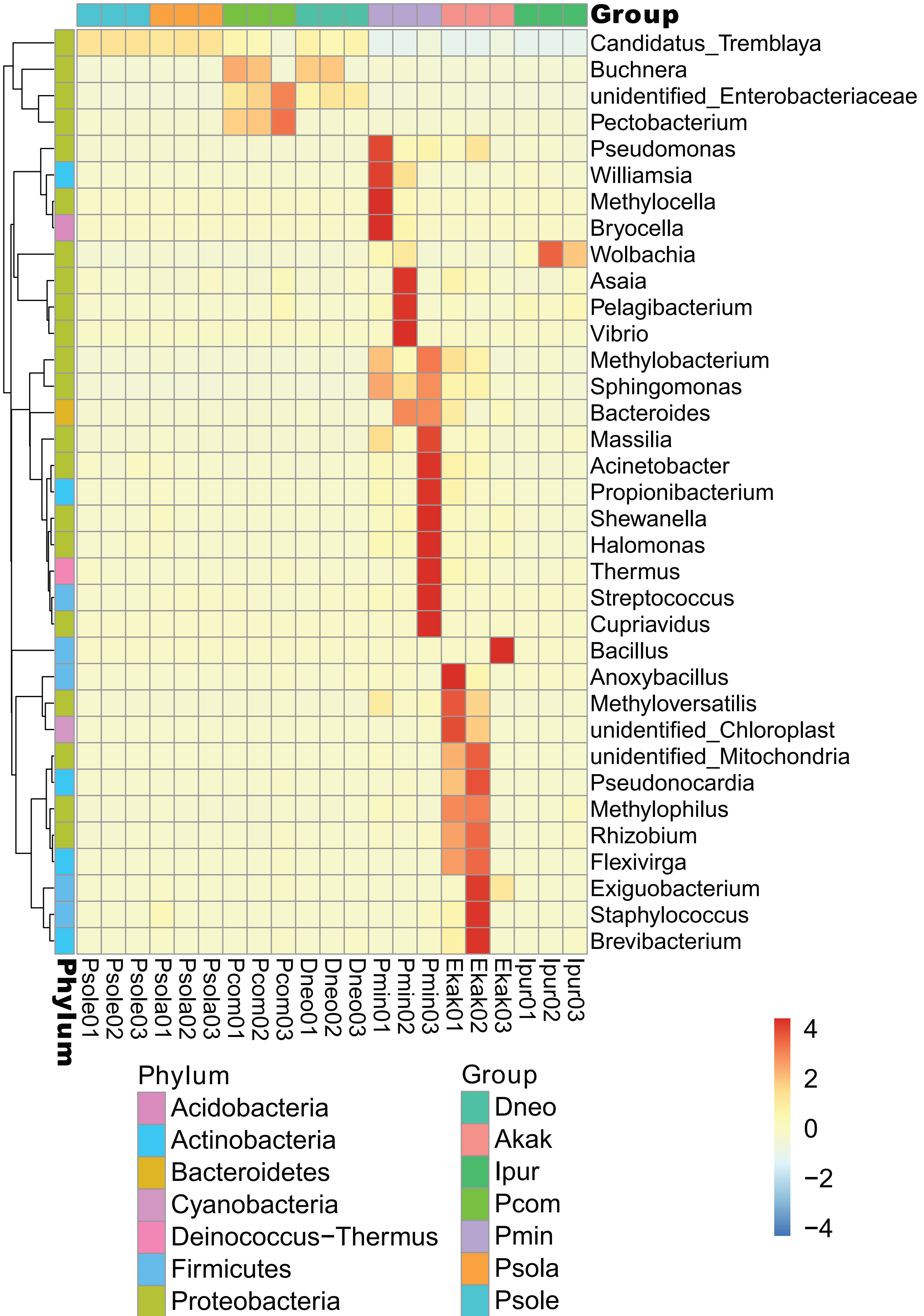
Group
Candidatus_Tremblaya
Buchnera
unidentified_Enterobacteriaceae
Pectobacterium
Pseudomonas
Williamsia
Methylocella
Bryocella
Wolbachia
Asaia
Pelagibacterium
Vibrio
Methylobacterium
Sphingomonas
Bacteroides
Massilia
Acinetobacter
Propionibacterium
Shewanella
Halomonas
Thermus
Streptococcus
Cupriavidus
Bacillus
Anoxybacillus
Methyloversatilis
unidentified_Chloroplast
unidentified_Mitochondria
Pseudonocardia
Methylophilus
Rhizobium
Flexivirga
Exiguobacterium
Staphylococcus
Brevibacterium
Phylum
Psole01
Psole02
Psole03
Psola01
Psola02
Psola03
Pcom01
Pcom02
Pcom03
Dneo01
Dneo02
Dneo03
Pmin01
Pmin02
Pmin03
Ekak01
Ekak02
Ekak03
Ipur01
Ipur02
Ipur03
4
2
0
−2
−4
Phylum
Acidobacteria
Actinobacteria
Bacteroidetes
Cyanobacteria
Deinococcus−Thermus
Firmicutes
Proteobacteria
Group
Dneo
Akak
Ipur
Pcom
Pmin
Psola
Psole

Figure S5

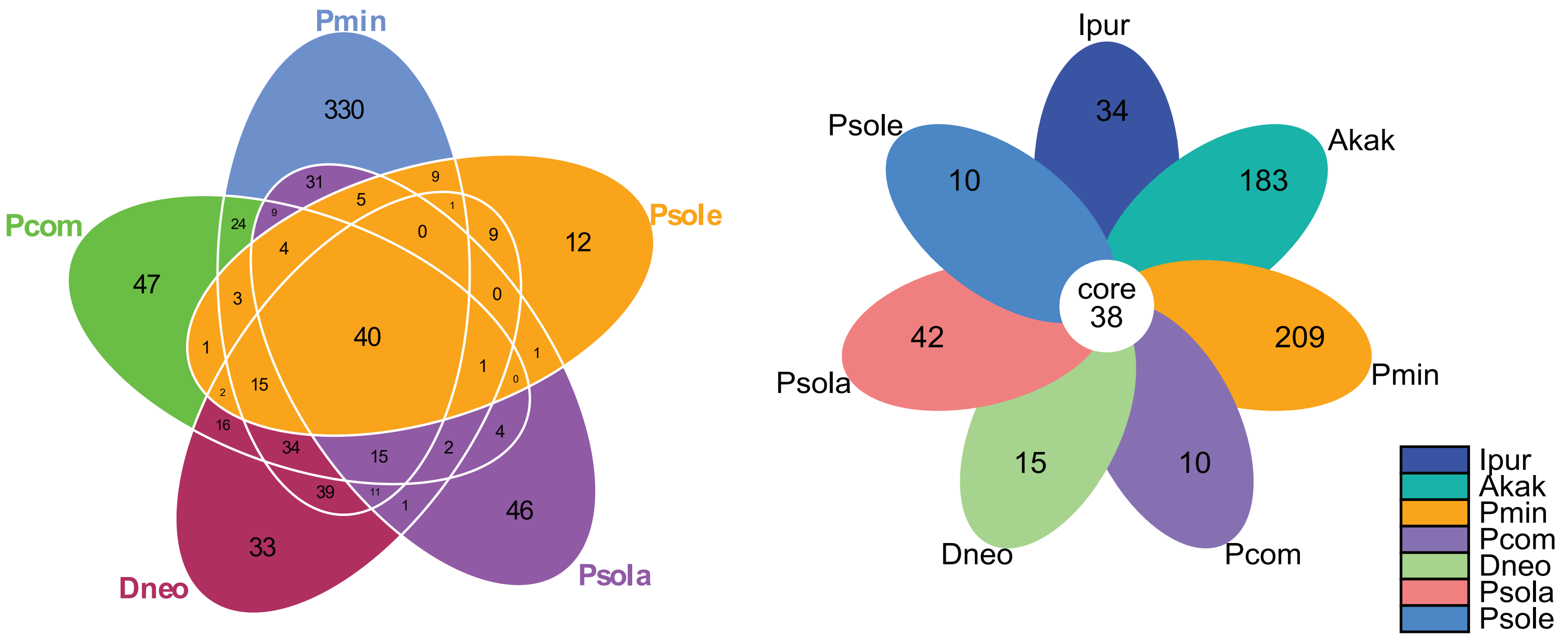
Pmin
330
Pcom
47
Psole
12
Dneo
33
Psola
46
40
31
5
9
1
9
24
9
4
0
3
0
1
1
1
15
0
2
16
34
15
2
4
39
11
1
Ipur
34
Psole
10
Akak
183
core
38
Psola
42
209
Pmin
15
10
Dneo
Pcom
Ipur
Akak
Pmin
Pcom
Dneo
Psola
Psole

(A)

(B)
